# Different HIV Drugs Cause Different Lipid Profiles

**DOI:** 10.1371/journal.pmed.0010030

**Published:** 2004-10-19

Nevirapine and efavirenz are the most commonly prescribed of the class of antiretroviral drugs called non-nucleoside reverse transcriptase inhibitors (NNRTIs). Efavirenz has the advantage of once-daily dosing. In a recent study called the 2NN study (Lancet 363: 1253–1263), it appeared to be only marginally superior to nevirapine in terms of clinical success and virological suppression. Van Leth and colleagues have now shown that while nevirapine and efavirenz both raise high-density lipoprotein (HDL) cholesterol (the “good” type of cholesterol), the overall lipid profile is better with nevirapine than with efavirenz.

“These data suggest that nevirapine may be preferable to efavirenz in HIV-infected adults with other cardiovascular risk factors,” says the study's academic editor, Andrew Carr of St. Vincent's Hospital in Darlinghurst, Australia. “However, perceived cardiovascular risk is only one factor that would affect the choice between these two drugs.”

Van Leth and colleagues prospectively analyzed the lipids of patients enrolled in the 2NN study, a randomized, open-label efficacy study that included adults with HIV who had never been on antiretroviral drugs. All patients were given stavudine and lamivudine and were then randomized into three treatment groups: nevirapine, efavirenz, or both.

For the lipid analysis, which was preplanned, the researchers included only the nevirapine and efavirenz groups (417 and 289 patients, respectively). This was because the 2NN study showed that simultaneous use of nevirapine and efavirenz should be avoided—the combination is associated with increased toxicity without increased efficacy. The increase in HDL cholesterol was significantly higher with nevirapine than with efavirenz. There was a decrease in the ratio of total cholesterol to HDL cholesterol with nevirapine and an increase with efavirenz.

The study does not prove, however, that the rise in HDL cholesterol seen with NNRTIs (especially nevirapine) actually leads to a reduction in coronary heart disease. “There are no vascular functional data,” says Carr, “or clinical vascular endpoint data that confirm that the statistically significant lipid differences observed are clinically significant.”

The study was funded by Boehringer Ingelheim, the manufacturer of nevirapine. The authors clearly state that the company had “a nonbinding input on issues of study design and analyses” but it had “no influence on reporting of the data or the decision to publish.”

Despite its limitations, van Leth and colleagues' study “moves clinicians and patients away from ‘one-size-fits-all’ antiretroviral therapy,” says Carr. “It takes us further along the path of choice of antiretroviral therapy being individualized according to other patient comorbidities and risk factors, as well as therapy simplicity and side effects.”

